# Low back pain in military recruits in relation to social background and previous low back pain. A cross-sectional and prospective observational survey

**DOI:** 10.1186/1471-2474-6-25

**Published:** 2005-05-26

**Authors:** Lise Hestbaek, Kristian Larsen, Flemming Weidick, Charlotte Leboeuf-Yde

**Affiliations:** 1 The Back Research Center, University of Southern Denmark and Hospital of Fynen, Lindevej 5, 5750 Ringe, Denmark; 2 The Medical Research Unit, County of Ringkjøbing, Amtsrådhuset, Torvet, 6950 Ringkøbing, Denmark; 3 Private practice, Herningvej 23, 7270 Stakroge, Denmark; 4 The Back Research Center, University of Southern Denmark and Hospital of Fynen Lindevej 5, 5750 Ringe, Denmark

## Abstract

**Background:**

Traditionally, studies on the etiology of low back pain have been carried out in adult populations. However, since low back pain often appears early in life, more research on young populations is needed. This study focuses on the importance of social background factors and previous low back pain in the development of low back pain in military recruits.

**Methods:**

During a three-month period, Danish military recruits with different social backgrounds live and work under the same conditions. Thus, there is an opportunity to investigate the influence of social background on the development of low back pain, when persons are removed from their usual environment and submitted to a number of new stressors. In addition, the importance of the recruits' previous low back pain history in relation to low back pain during military service was studied. This was done by means of questionnaires to 1,711 recruits before and after this three-month period.

**Results:**

Sedentary occupation was negatively associated with long-lasting low back pain (>30 days during the past year) at baseline with an odds ratios of 0.55 (95% CI: 0.33–0.90). This effect vanished during service. Having parents with higher education increased the risk of low back pain during service (OR: 1.9;1.2–3.0, for the highest educated group), but not of the consequences (leg pain and exemption from duty), whereas high IQ decreased the risk of these consequences (odds ratios as low as 0.2;0.1–0.8 for exemption from duty in the group with highest IQ). Long-lasting low back pain prior to service increased the risk of long-lasting low back pain (OR: 4.8;2.1–10.8), leg pain (OR: 3.3;1.3–8.3) and exemption from duty during service (OR: 5.9;2.4–14.8).

**Conclusion:**

Sedentary occupation is negatively associated with low back pain at baseline. This protective effect disappears, when the person becomes physically active. For predicting trouble related to the low back during service, the duration of low back pain prior to service and IQ-level are the most important factors.

## Background

Low back pain (LBP) is a very common ailment in the Western World and musculoskeletal disorders are leading causes of long-term sick leave [1]. Thus, it has a major social and economic impact on society. Although elimination of LBP is only wishful thinking, prevention of chronicity may be within the scope of reality – with obvious gains for society. To obtain this goal, a thorough understanding of the etiology is necessary. Presently, etiological research has a broad approach to LBP, based on the bio-psycho-social model and several studies have been performed in which risks associated with physical characteristics [2], psychological characteristics [3], lifestyle factors [4], employment [5-8], social factors [9,10] and genetic components [11,12] were investigated. However, these studies are difficult to interpret due to the close relationship between social factors, intellectual capacity, coping strategies, education, profession etc., causing a mesh of interactions and confounding.

Traditionally such studies have been carried out on working-age populations, but there is growing evidence that LBP has an earlier onset than hitherto thought. In a study of Danish twins, aged 12 to 41, the one-year prevalence of LBP was found to rise from 7% in those aged 12 to above 50% in those aged 22 and to increase only slightly thereafter to reach 56% in those at the age of 41 [13]. Furthermore, earlier studies have shown that the risk of LBP at age 30 is significantly increased for individuals with LBP at age 18 [14]. Obviously, to obtain a thorough understanding of the etiology of LBP, young populations should be investigated.

The Danish Army has a system of mandatory conscription for six months of service. Usually, there is an intake in August and one in January, with most high school graduates commencing in August after finishing high school, whereas the January-intake mainly consists of those with no academic education beyond primary school. However, due to special circumstances, there was no intake in January 2000 and therefore the August-intake of that year represented the social variety of the Danish society, as reflected by education. During the first three months of service all the recruits receive the same training, housing etc. Therefore, we had a unique opportunity to investigate the development of LBP in young persons (primarily males) of different educational backgrounds, who were fit for military service, at a time when they were submitted to the same living and working conditions. This made it possible to investigate the impact of various social background factors on the occurrence of LBP, without results being confounded by differences in lifestyle and type of work. By surveying these conscripts on their first day of enrollment, we can estimate how their background influences their prevalence of LBP, reflecting the influence of their usual environment. After three months of being subjected to unusual stressors in a different environment, like performing physically demanding tasks whilst being deprived of their normal personal freedom, we can estimate how the occurrence of LBP is influenced by their social background as well as their baseline LBP-status. Thus, the present study has two objectives:

1) To investigate possible associations between social background factors and the prevalence of LBP in young persons, both in their usual environment and in a new setting.

2) To identify possible predictors, both social and LBP-related, for reporting LBP during military service.

## Methods

### Subjects

During the year of 2000, 2,343 persons, aged 18–24, were enrolled as military conscripts at 15 locations across Denmark. Upon reporting for duty, the recruits had a medical examination and during the course of this, a research questionnaire was administered. The medical officer filled in the first part, which contained the army's own information: personal identification number, years of schooling, the score from the military's intelligence test, and type of work. The second part was filled in by the conscripts themselves and contained questions relating to their LBP-history and the education of their parents. After three months of basic training the conscripts were given different tasks, thus conditions were no longer the same for all and our follow-up period ended. At this time they were given a second questionnaire relating to LBP during their first three months of service. An information letter accompanied the questionnaires, explaining the purpose of the project and assuring the confidentiality of the informant. It was stressed, that the information would not be available to the army, as only staff at the Medical Research Unit in the county of Ringkøbing would have access to the information.

### Variables

#### Social background variables

The following background information was collected routinely by the military on all potential recruits:

• Years of school: Years of schooling from 1^st ^grade till joining the military. Treated as a continuous variable.

• IQ-score: Result of an intelligence test performed when examined for liability for military service. Divided into three categories with cutpoints close to the 25- and the 75-percentile (low, medium, high) and treated as a categorical variable.

• Occupation: Each profession has its own code in the military. These codes were translated and dichotomized into sedentary or manual work.

The questionnaires contained the following information:

Parents' education: The level of the highest educated parent. Choices were: grade school only, high school level, or >12 years (university/other type of higher education).

#### LB-related variables

• LBP: Questionnaire 1: Pain or discomfort in the lower back during the past year (yes/no). Questionnaire 2: Pain or discomfort in the lower back during military service (yes/no). This was accompanied by a drawing, showing the lower back as the area between the gluteal folds and the 12^th ^ribs.

• Duration: Questionnaire 1: Number of days with LBP during the past year. Questionnaire 2: Number of days with LBP during military service. This was treated as a categorical variable (0 days, 1–7 days, 8–30 days, >30 days) and as a dichotomous variable (long-lasting LBP (LLBP) = LBP>30 days, yes/no).

• Leg pain: Questionnaire 1: Leg pain radiating from the low back during the past year (yes/no). Questionnaire 2: Leg pain radiating from the low back during military service (yes/no).

• Exemption: Questionnaire 2: Number of days exempted from duty due to LBP during the follow-up period. Due to the small number of exemptions, this was formed into a dichotomous variable (Exemption yes/no).

The questions of one-year prevalence of LBP, duration of LBP, and leg pain were modeled on the Nordic low back pain questionnaire [15] of which the LBP-questions have been validated in previous studies [16,17].

### Analysis

The analysis is divided into two parts: 1) a cross-sectional study investigating associations between LBP and the described social variables (IQ, years of schooling, occupation and parents' education) and 2) a three-months follow-up study investigating how LBP during military service is related to the social background factors and baseline low back related (LB-related) factors: LBP, long-lasting LBP and radiating pain to the leg. All outcome variables were analyzed seperately for the total sample, i.e. leg pain against no leg pain, regardless of LBP-status. Since the social background variables are closely related, they were all tested for interactions by means of the Mantel-Haenzel homogeneity test.

All analyses were performed with Stata 7.0. Statistical significance was defined at the 5%-level. However, since multiple associations were tested, one might choose to be restrictive in the interpretations of significance. Therefore, statistical significance has been indicated in the relevant tables at the 1%-level as well.

#### Cross-sectional study

The baseline outcome variables were analyzed with respect to the social background variables by means of multivariate logistic regression. Following multivariate analyses, forward exclusion was applied to decide which variables to keep in the models for the different outcome variables. The variables were kept in the models if they were statistically significant or if they had a bearing on the estimates of the variables that were statistically significant in the multivariate analyses. They were included if they changed the estimates of the statistically significant variables by 25% or more, if they changed the corresponding p-values with 25% or more, or if they changed the significance status of the relevant estimates. Results are presented as odds ratios with 95% confidence intervals (CI).

#### Follow-up study

The same type of regression-analyses were used to analyze the follow-up outcome measures with respect to the social background variables. The most relevant baseline LB-related variable(s) was/were determined through univariate logistic regression, and a final model for predicting LB-related trouble during military service was constructed by combining social and LB-related variables.

#### Post-hoc analyses

Due to the low response rate at baseline, it was decided to compare the prevalence rates of the baseline LBP-variables to those of the background population. This was done by means of data from the Danish Twin Register, which previously has been shown to be representative of the general Danish population and is described in detail elsewhere [18]. The twins had answered a questionnaire containing the same LBP-questions in 1994. Therefore, a nested cohort of 18–22 year old males was selected for comparison to the soldiers in the present study.

Finally, the consequence in relation to exemption from duty were demonstrated by calculating the reduction in intake of recruits and the corresponding reduction in exemptions, if the identified significant factors were to be used as part of the inclusion criteria for military service. In this analysis, only subjects who have answered all the relevant questions are included.

## Results

### Response rate

Of the originally planned enrollment of 2,343 individuals, 1,711 (73%) returned the baseline questionnaire with valid answers to the LBP-questions. The follow-up response rate is 58% (985/1711). The numbers of valid responses to the individual variables are shown in Table 1.

**Table 1:** Number of responses to the questionnaires.

**Years of school**.						n: 1,657
**IQ**	Low score	Medium score		Highest score		n
	351	682		406		1,439
						
**Occupation**	Sedentary occupation		Manual occupation			n
	840		807			1,647
						
**Parental education**	Grade school only	High school		>12 years of education		n
	474	521		461		1,456
						
**LBP**		Yes		No		n
	Questionnaire 1	712		999		1,711
	Questionnaire 2	345		639		985
						
**Duration:**		0 days	1–7 days	8–30 days	>30 days	n
	Questionnaire 1	948	243	259	158	1,608
	Questionnaire 2	637	140	119	49	945
						
**Leg pain**		Yes		No		n
	Questionnaire 1	82		1589		1,671
	Questionnaire 2	66		912		982
						
**Exemption**	Yes		No			n
	65		806			871

### Gender and age

The study sample is predominantly male with only 4% females. The mean age is 20.57 years with a standard deviation of 2.16 years, ranging from 18 to 30 years of age.

### Quality of data

Double entry of a 10%-subsample revealed an error-rate of less than 0.6%, which we consider satisfactory.

### External validity (post-hoc analysis)

The one-year prevalence rates of LBP, leg pain and long-lasting LBP (LLBP) are shown in Table 2 for 18–22 year old male twins from the Danish Twin Register and for the soldiers at baseline of the present survey.

**Table 2:** One-year prevalence rates with 95% confidence intervals of the soldiers at baseline in the present study and in an age-matched cohort, representative of the background population (18–22 year old male twins, surveyed in 1994).

Cohort	LBP	LLBP	leg pain
2,213 twins	43% (41–45%)	7% (6–9%)	5% (4–6%)
1,711 soldiers	42% (39–44%)	7% (6–9%)	5% (4–6%)

The present sample of soldiers is perfectly comparable to an age- and almost sex-matched sub-sample from the Danish Twin Registry with regard to the outcome variables: one year prevalence of LBP, long-lasting LBP and leg pain.

### Analysis of non-responders at follow-up

There was no difference in responders and non-responders at follow-up with regard to any of the social background factors or the one-year prevalence of LBP at baseline. However, non-responders had more days with LBP during the past year than the responders (mean 16.11 (SD 59.45) and mean 10.74 (SD 37.24), respectively, p = 0.000), and there was also a statistically significant difference in leg pain (p = 0.027) with 6% (5–8%) of non-responders and 4% (3–5%) of responders complaining of leg pain, indicating that the LBP in non-responders might be more severe in nature.

### Interactions between social background variables

All the background variables were positively interrelated (p ≤ 0.08), but tests for homogeneity showed no interactions (p ≥ 0.72).

### Cross-sectional analysis

#### Model for associations between LB-related variables at baseline and social background variables

The statistically significant factors found in the multivariate analysis were parents' education for LBP, occupation and parents' education for long-lasting LBP, but none for leg pain. The forward exclusion method left parents' education, sedentary job and IQ in the model for LBP and all four variables in the model for long-lasting LBP (removing years of schooling or IQ both lead to a decrease in the p-value of the effect-estimate of occupation of more than 25%).

Having a sedentary job showed a reduced association with LBP. Although this was not statistically significant for simply having LBP, it was highly significant for long-lasting LBP.

Having the highest educated parent at high school level lead to a statistically significant higher prevalence of LBP and long-lasting LBP whereas the findings for those with a parent with >12 years of education were not statistically significant and no gradient across groups was detected. The results are presented in Table 3.

**Table 3:** Final model for associations between LBP-variables at baseline and social background variables (odds ratios with 95% confidence intervals). Significant findings are indicated with bold typing.

N = 1708	LBP n = 710	LLBP n = 157
IQ-score low	1.00	1.00
IQ-score medium	1.09 (0.81–1.48)	1.09 (0.69–1.73)
IQ-score high	1.19 (0.84–1.68)	0.95 (0.54–1.68)
Sedentary occupation	0.84 (0.65–1.08)	0.55 (0.33–0.90)*
Parents grade school	1.00	1.00
Parents high school	1.39 (1.05–1.84)*	1.67 (1.05–2.64)*
Parents >12 years education	1.23 (0.93–1.64)	1.33 (0.82–2.17)
Years of school	-	0.98 (0.89–1.08)

*: p < 0.05, **: p < 0.01

### Follow-up analysis

#### Model for associations between social background variables and LBP during military service

The multivariate analysis showed no statistically significant findings for long-lasting LBP. Parents' education was found to be statistically significant for LBP during service with an increasing gradient, i.e. higher education associated with higher prevalence of LBP. IQ was inversely related to both leg pain and exemption during service with decreasing gradients, i.e. the higher the score, the lower the risk. Years of schooling could be excluded from the model for LBP and leg pain without any bearing on the estimates, and type of occupation could be excluded from the model for exemption from duty. The exact estimates are shown in Table 4.

**Table 4:** Final model for associations between social background variables and LBP during military service (odds ratios with 95% confidence intervals). Statistically significant findings are indicated with bold typing.

N = 982	LBP n = 345	Leg pain n = 66	Exemption n = 65
IQ-score low	1.00	1.00	1.00
IQ-score medium	1.02 (0.68–1.52)	0.72 (0.37–1.42)	0.56 (0.27–1.17)
IQ-score high	0.99 (0.64–1.53)	0.33 (0.13–0.88)*	0.26 (0.09–0.79)*
Parents grade school	1.00	1.00	1.00
Parents high school	1.58 (1.06–2.34)*	1.25 (0.62–2.54)	1.17 (0.54–2.55)
Parents >12 years education	1.81 (1.20–2.72)**	1.19 (0.56–2.49)	1.22 (0.54–2.76)
Sedentary occupation	0.84 (0.59–1.20)	0.69 (0.36–1.33)	-
Years of school	-	-	0.89 (0.71–1.10)

*: p < 0.05, **: p < 0.01

#### Model for associations between LB-related trouble at baseline and LB-related trouble during military service

All the LB-related variables were significantly associated with all the outcome variables. We decided to use number of days with LBP during the past year rather than LBP at all during past year to obtain more detailed analyses. There is a high correlation between baseline leg pain and the outcome variables (73 of the 82 (89%) persons with leg pain also had LBP), and adding leg pain to the models does not alter the estimates significantly. With regard to leg pain at follow-up, baseline leg pain is the most important factor. The results of the bivariate logistic regression analyses are shown in Table 5.

**Table 5:** Associations between baseline LB-related variables and LBP during military service (odds ratios with 95% confidence intervals) as assessed by univariate logistic regression analyses. Statistically significant findings are indicated with bold typing.

N = 982	LBP n = 345	Leg pain n = 78	LLBP n = 56	Exemption^1 ^n = 74
Baseline LBP	3.52** (2.68–4.63)	1.71** (1.15–2.54)	1.58* (0.01–1.28)	2.89* (1.70–4.89)
Baseline leg pain	2.22* (1.15–4.30)	8.78** (4.23–8.21)	4.92** (2.03–11.89)	3.61** (1.50–8.69)
0 days with LBP previous year	1.00	1.00	1.00	1.00
1–7 days with LBP previous year	2.04** (1.39–2.99)	0.90 (0.36–2.24)	1.11 (0.44–2.84)	1.96 (0.95–4.06)
8–30 days with LBP previous year	5.42** (3.63–8.08)	2.57** (1.28–5.14)	2.47* (1.14–5.32)	1.44 (0.63–3.30)
>30 days with LBP previous year	6.45** (3.86–10.79)	5.51** (2.73–11.12)	4.80** (2.13–10.84)	6.13** (3.05–12.50)

*: p < 0.05, **:p < 0.01

#### Final models for predicting trouble related to LBP during military service combining social background variables and LB-related baseline-variables

When the models for associations between social background and LB-related trouble during military service were combined with LB-related baseline variables, the following models could be derived:

#### LBP during military service

The model for the association between social background variables for LBP during service included IQ and parents' education. When this was combined with the number of days with LBP the year prior to service, parents' education remained statistically significant with an increase in education resulting in an increase in odds ratio. Number of days with LBP showed the strongest correlation with odds ratios above 6. IQ-level could not be excluded from the model without altering the significance level of parents' education

#### Long-lasting LBP during military service

None of the social background variables had significant influence on the development of long-lasting LBP, thus only the LB-related baseline variable, duration of LBP, was included. The effect of LBP-duration was statistically significant, demonstrating a positive association (OR = 4.8 for LBP>30 days at baseline).

#### Leg pain related to LBP during military service

IQ, parents' education and type of occupation were included in the model for leg pain during service. Adding leg pain during the year prior to service and number of days with LBP during the same period, leave the same variables as significant, but excluding parents' education from the model would change the significance of both IQ and duration of LBP. A high IQ-level showed a negative association with leg pain during service, whereas long duration of LBP and previous leg pain both showed positive associations (OR = 3.3 and 3.1, respectively).

#### Exemption from duty due to LBP during military service

Including the relevant factors in the model (IQ, parents' education, years of schooling and number of days with LBP), left IQ and number of days with LBP to be significant. However, once more, excluding parents' education or years of schooling, changed the significance level for IQ and it was therefore kept in the model. A high IQ lowered the risk of exemption (OR = 0.2) whereas long-lasting LBP the previous year increased the risk (OR = 5.9).

The results of the final models for all outcome measures are shown in Table 6.

**Table 6:** The final models for outcome measures during service, combining social and physical factors (odds ratios with 95% confidence intervals). Statistically significant findings are indicated with bold typing.

N = 982	LBP n = 345	LLBP n = 56	Leg pain n = 66	Exemption^1 ^n = 65
IQ-score low	1.00	-	1.00	1.00
IQ-score medium	1.14 (0.73–1.79)	-	0.75 (0.35–1.61)	0.56 (0.25–1.25)
IQ-score high	1.00 (0.61–1.63)	-	0.24 (0.08–0.74)**	0.24 (0.08–0.76)*
Parents grade school	1.00	-	1.00	1.00
Parents high school	1.56 (1.01–2.42)*	-	1.05 (0.48–2.27)	1.14 (0.48–2.71)
Parents >12 years education	1.90 (1.21–2.99)**	-	1.06 (0.47–2.38)	1.40 (0.59–3.35)
Sedentary occupation	-	-	0.87 (0.42–1.79)	-
Years of school	-	-	-	0.91 (0.72–1.14)
Baseline Leg pain	-	-	3.14 (1.13–9.85)*	-
0 days with LBP previous year	1.00	1.00	1.00	1.00
1–7 days with LBP previous year	2.31 (1.49–3.59)**	1.11 (0.44–2.84)	0.79 (0.28–2.22)	2.33 (0.94–5.79)
8–30 days with LBP previous year	6.09 (3.78–9.84)**	2.47 (1.14–5.32)*	2.52 (1.28–6.20)**	1.78 (0.64–4.94)
>30 days with LBP previous year	6.18 (3.42–11.17)**	4.80 (2.13–10.84)**	3.28 (1.31–8.25)**	5.93 (2.37–14.83)**

*: p < 0.05, **:p < 0.01

### Consequences

For practical purposes, in the process of selecting military recruits for army, the outcome of interest is exemption from duty. For this outcome, the most interesting factors, of the ones investigated in this study, are the number of days with LBP during the year prior to service and the IQ-level. Table 7 presents an estimation of the reduction in the number of soldiers exempted from duty and the corresponding reduction in intake if various combinations of those two factors were used as inclusion criteria. The practical significance must then be judged in the actual situation, for example the number of available potential recruits.

**Table 7:** Consequence in reduction of exemptions and intake-size when excluding subgroups of potential recruits from service, on basis of the two factors found to influence the rate of exemption from duty significantly (IQ-score and duration of prior LBP).

**Included**	**# exempted from duty**	**Total intake**
Full sample	45 (100%)	664 (100%)
IQ-score=high	8 (18%)	187 (28%)
IQ-score=high or medium	29 (64%)	512 (77%)
LBP-days<30	34 (76%)	607 (91%)
IQ-score=high & LBP-days<30	6 (13%)	169 (25%)
IQ-score=high or medium & LBP-days<30	21 (47%)	467 (70%)

## Discussion

The major advantage of our cohort is that people of similar age from backgrounds reflecting the variety of society are all exposed to the same living and working conditions for the same period of time when doing their military service. Obviously, only subjects fit for military service are included, thus excluding the most intellectually and physically disadvantaged. It has, however, previously been established, that the prevalence of LBP in Danish conscripts is similar to the rest of the Danish population of that age [19]. This we confirmed, as the present cohort does have similar prevalence rates of the LBP-variables as a comparable background population. The major problem of our study is the low response rate (58%). There might have been whole platoons/units (rather than individuals) that did not return the questionnaires. However, we were unable to confirm this, as all the questionnaires were collected in one box upon arrival at the research centre and the place of duty was not stated in the questionnaire. Furthermore, the personal identification number of each soldier was used to combine the first and second questionnaires. A large number of soldiers returned the second questionnaire without stating this number, therefore leaving us with a large number of un-identifiable, although otherwise completed, questionnaires. Thus, most of this is likely to reflect an administrative error (whole platoons not returning the questionnaires) and poor design of the questionnaires (the first and second questionnaire could not be linked) and therefore one would not expect any disease-specific bias as a result of non-response. This is also confirmed by the similarity of prevalence rates between our study sample and the sample from the Danish Twin Register. Anyway, the problem of non-responders having more severe LBP at baseline still remains. This could be related to termination of service prior to follow-up due to LBP of some individuals with a high level of baseline-LBP. Unfortunately, we did not have access to such information. In the protocol, the army staff was asked to administer the second questionnaire to the recruit in case of rejection during service, but this did not happen in any instance. This bias does not directly influence the results relating to the social background variables, but it might weaken the results relating to baseline LBP-status with associations actually being stronger than our findings indicates, if the rejected recruits were the ones with the more severe LBP-symptoms at baseline.

As for the influence of social background factors on the presence of LBP at baseline in our population, we found a statistically significant increase in odds ratios if parents belonged to educational group 2 (high school-level). This is an illogical finding that might be an effect of multiple testing and since there is no gradient from the lowest to the highest group, the practical significance of this result is limited. However, when analyzing the occurrence of LBP and related trouble during military service, parents' education does show an increasing gradient for LBP lending support to the credibility of the baseline-findings. A previous study found no association between parents' education and back pain in Danish adolescents [20].

The most important factor for LBP at baseline was found to be type of occupation. This is in line with a previous study, showing sedentary occupations to have a "protective effect" on LBP [21]. Interestingly, there was no effect of pre-service occupation on LBP and related troubles during military service – those with sedentary occupations were as likely as the rest to develop LBP, when they were exposed to the same stress. This implies that the association with a sedentary occupation is direct, rather than a proxy of intelligence level, social class or other similar confounders/interactions.

Intelligence level, as measured by the army's IQ-test, did not influence any of the LBP-variables at baseline, and did not influence the occurrence of LBP during service. A high test-result did, however, limit the occurrence of leg pain and exemption from duty. Both sedentary occupation and parents' education are strongly associated with intelligence level (p = 0.000 and p = 0.008, respectively). Thus, by taking our results a step further, it could be speculated that the group with above-average intelligence, well-educated parents and a sedentary occupation has a low occurrence of LBP at baseline, an increased risk of LBP during the physical demands of service, but seem to suffer less from the consequences (leg pain and exemption from duty). This might be a sign of better coping strategies in this group. Coping strategies as they relate to dealing with chronic pain has been a focus of research in recent years [22-24], but coping abilities as part of the etiology of back pain has only received limited attention yet. In a recent study of a general population, high levels of passive coping were found to be associated with disabling neck and back pain [25].

When the LBP-variables at baseline are added to the model for LBP-related trouble during service, the duration of LBP the previous year is the important variable. LBP in itself does not predict LBP during service but a longer duration of LBP at baseline increased the risk of long-lasting LBP, leg pain and exemption from duty during the study period. This might indicate that short-lasting LBP is more co-incidental and a normal life experience. This is consistent with the literature, where the duration of LBP is considered to be an important prognostic factor [26-29]. Furthermore, we showed that leg pain at baseline is a strong predictor for continued leg pain.

As a screening tool to predict which recruits will develop LBP during military service, models containing the investigated variables are not sufficiently accurate. If used to exclude vulnerable subjects from military service, many would be excluded unnecessarily.

## Key points

• Having a sedentary occupation is negatively associated with long-lasting LBP and leg pain in this population, irrespective of intelligence level and education.

• Having parents with a high education may increase the risk of developing LBP during military service, but not of its consequences.

• A high IQ seems to protect against leg pain and exemption from duty during military service.

• Long-lasting LBP at baseline increases the risk of long-lasting LBP, leg pain and exemption during the course of duty.

• The predictive value of the investigated factors is too low to be used as a screening tool for the military.

## Conclusion

With regard to the social variables (IQ, occupation, and parents' education), type of occupation is the most important factor at baseline with a sedentary occupation having a negative association with LBP. This protective effect disappears, when the person actually enrolls and becomes more physically active. Then, a higher level of parents' education seems to predispose for simple LBP, whereas a high intelligence level is a protective factor for the development of leg pain and exemption from duty.

With regard to the LBP-related variables (LBP at all, long-lasting LBP, and leg pain), longer duration of LBP during the previous year increases the risk of all LBP-related problems during military service. Finally, the presence of leg pain at baseline predicts leg pain during service.

Our findings provide important information in relation to etiology, but a suitable screening instrument will require further refinement.

## Competing interests

The author(s) declare that they have no competing interests.

## Authors' contributions

LH participated in the design and coordination of the study, double-entered a sub-sample of data, performed the statistical analyses and drafted the manuscript. KL participated in the design and coordination of the study and confirmed the statistical analyses. FW provided contact to the army practitioners and participated in the coordination of the study. CL-Y participated in the design of the study and in drafting the final manuscript. All authors read and approved the final manuscript.

## Pre-publication history

The pre-publication history for this paper can be accessed here:


